# Graphene Oxide-Doped CNT Membrane for Dye Adsorption

**DOI:** 10.3390/nano15110782

**Published:** 2025-05-22

**Authors:** Mariafrancesca Baratta, Fiore Pasquale Nicoletta, Giovanni De Filpo

**Affiliations:** 1 Department of Chemistry and Chemical Technologies, University of Calabria, 87036 Rende, Italy; 2 Department of Pharmacy, Health and Nutritional Sciences, University of Calabria, 87036 Rende, Italy; fiore.nicoletta@unical.it

**Keywords:** water pollutants, carbon nanotubes, buckypapers, graphene oxide, dyes

## Abstract

Recently, graphene oxide (GO) has been largely investigated as a potential adsorbent towards dyes. However, the major obstacle to its full employment is linked to its natural powder consistence, which greatly complexifies the operations of recovery and reuse. With the aim to overcome this issue, the present work reports on the design of GO-modified carbon nanotubes buckypapers (BPs), in which the main component, GO, is entirely entrapped in the BP grid generated by CNTs for the double purpose of (a) increasing adsorption performance of GO-BPs and (b) ensure a fast process of regeneration and reuse. Adsorption experiments were performed towards several dyes: Acid Blue 29 (AB29), Crystal Violet (CV), Eosyn Y (EY), Malachite Green (MG), and Rhodamine B (RB) (C_i_ = 50 ppm, pH = 6). Results demonstrated that adsorption is strictly dependent on the charge occurring both on GO-BP and dye surfaces, observing great adsorption capacities towards MG (493.44 mg g^−1^), RB (467.35 mg g^−1^), and CV (374.53 mg g^−1^), due to the best coupling of dye cationic form with negative GO-BP surface. Adsorption isotherms revealed that dyes capture onto GO-BPs is thermodynamically favored (ΔG < 0), becoming more negative at 313 K. Kinetic studies evidenced that the process can be described through a pseudo-first-order model, with MG, RB, and CV exhibiting the highest values of k_1_. In view of these results, the following trend in GO-BP adsorption performance has been derived: MG ≈ RB > CV > AB29 > EY.

## 1. Introduction

Together with water scarcity, water pollution represents one of the biggest threats and one of the most difficult challenges of this century. As evidence of this, the latest UNO Report on the status of achievement of Sustainable Development Goals contained in the 2030 Agenda states that progress in G6 Goal (“Ensure availability and sustainable management of water and sanitation for all”) “remains insufficient, despite some improvements. At the current speed, in 2030, 2 billion people will still live without safely managed drinking water, 3 billion without safely managed sanitation and 1.4 billion without basic hygiene services” [1]. One of the major difficulties in resolving the problem of water pollution is indeed the wide variety and complexity of contaminants ending up in wastewater, making current wastewater treatment technologies inefficient and increasingly obsolete. Among the unlimited possibilities of organic and inorganic pollutants found in water, dyes and pigments stand out for their huge amount and diversity. This strong occurrence is the result of application of dyes, both natural or synthetic, in a considerable part of anthropogenic activities, covering a lot of industry sectors, such as paper, leather, food, cosmetics, paint, and, above all, textiles. The latter, indeed, consumes more than 80% of global annual production of synthetic dyes, estimated to be around 800,000 tons per year [2], managing a textile market that, according to latest Market Report by IMARC Group, reached a value of USD 1065.6 Billion in 2024, and it is expected to increase by an additional 39% by 2033 [3]. Furthermore, most textile manufacturing operations, such as dyeing, finishing, tinting, sourcing, sizing, de-sizing, and printing, are detrimental, since huge volumes of textile waters produced during these processes and containing dyes not stuck on fabrics are often released into the environment without adequate control [2]. The risk associated with the occurrence of dyes in water includes multiple factors. Generally, dyes are not bio-degradable compounds, with chemical resistance to heat and light, acting as promotors in discoloration of water bodies [4]. Moreover, due to their ability to absorb and reflect sunlight, the amount of light reaching the photic zone is strongly reduced by dyestuff occurrence, causing an interference in the natural photosynthetic activity of aquatic flora [5]. Thirdly, it has been demonstrated that dyes can have a mutagenic and carcinogenic potential towards human health, leading to the development of diseases such skin and eye irritation, dermatitis, ovulation and spermatogenesis interference, problems to the central nervous system, and carcinogenesis [2,6,7]. In this tragic scenario, a prompt action aimed at changing the course of events is extremely required.

Currently, treatment of dyestuff wastewater involves the employment of different technologies: physical such as sedimentation and adsorption; chemical like flocculation, photodegradation, chemical oxidation, and electrochemical treatments; and biological [8,9,10,11,12,13]. With a lot of advantages in terms of process feasibility, large recovery percentages, and eco-friendliness, adsorption represents, above all, an indispensable tool in the treatment of textile water [14,15]. Along these lines, adsorption can further count on a wide range of nanomaterials of recent development, whose large surface area strongly contribute to increase process recovery performance [16]. This includes TiO_2_ nanoparticles, silicates, resins, MoS2 nanosheets, porous polymers, and carbon-based materials such carbon nanotubes (CNTs), activated carbon, graphene, and graphene oxide (GO) [16,17,18,19]. Speaking about the latter, GO turned out to be an excellent candidate for the removal of dyes from wastewater. Its high surface area and flat morphology, resembling a 2D honeycomb structure, fit perfectly with the planarity of most of dyes, originated by the intensive number of aromatic rings in their structures, thus favoring the instauration of hydrophobic interactions with which dyes can be recovered. Furthermore, GO chemical tunability, due to the presence of -OH and -COOH polar functional groups, can enhance and reinforce dye adsorption through the additional establishment of electrostatic interactions, thus contributing to the acceleration and improvement in the entire process [20]. However, two of the major drawbacks associated with the employment of GO in aqueous solutions are related to the aggregation phenomenon of GO flakes, which consistently reduces the availability of surface area for adsorption, and the nanometric dimensions of particles, which complicates adsorbent recovery, unless additional steps of centrifugation and filtration are performed, and its reuse.

On the other hand, carbon nanotubes, also possessing a large surface area that makes them excellent adsorbents, differ from GO substantially for their tubular structure. Thanks to this and to their high mechanical properties, CNTs can be opportunely handled in buckypaper (BP) membranes entirely made of carbon nanotubes, thus developing a new “system” contemporaneously benefiting from the advantages coming from CNTs (chemical stability, electrical conductivity, large surface area) with those of a membrane (porosity, self-standing ability) [17,21,22]. In addition, the preparation of BPs through vacuum-assisted filtration can count on a fast, simple, and environmentally friendly procedure by dispersing CNTs in eco-friendly solvents, like methanol or water, which can be recovered at the end of each step of filtration and reused. Recent developments in this field have shown that BP adsorption properties can be further improved through the incorporation of porous adsorbents exhibiting large surface areas, such as metal organic frameworks (MOFs) [23,24]. In this case, the incorporation of powder dopants into BPs is functional to increase adsorption capacity as much as possible to avoid recovery problems due to BP standing-alone properties, facilitating the entire process in terms of recycling and regeneration [25].

Starting from these premises, the aim of the present work is focused on the preparation of GO–buckypaper membranes (GO-BPs), enriched through the entrapment of large amounts of GO (75% *w*/*w*), in order to exploit the great adsorption potential of GO towards dyes without renouncing the mechanical stability and self-standing ability of BPs, thus ensuring an efficient and fast possibility of regeneration and reuse of pristine adsorbents. Five different dyes have been investigated in order to evaluate GO-BPs’ performance in terms of selectivity as well, showing that chemical tunability of GO is the key factor in the adsorption of these pollutants.

## 2. Materials and Methods

### 2.1. Materials and Reagents

SWCNTs-COOH (average D = 4–5 nm, L = 0.5–1.5 µm, bundle dimensions) and graphene oxide GO (15–20 sheets, 4–10% edge-oxidized) were both purchased from Sigma-Aldrich, Milan, Italy. Acid Blue 29 (AB29), Crystal Violet (CV), Eosyn Y (EY), Malachite Green (MG), and Rhodamine B (RB) were also purchased from Sigma-Aldrich, Milan, Italy. All chemicals were used as received.

### 2.2. Buckypaper (GO-BP) Preparation

Preparation of GO buckypaper membranes (GO-BPs) has been carried out while referring to the wet method technique, following a procedure similarly reported in our previous work [26]. 12.5 mg of SWCNTs-COOH were dispersed in 150 mL of methanol with 37.5 mg of graphene oxide, GO (GO:SWCNTs-COOH: 75:25 *w*/*w*%). After sonication (4 h) in an ultrasonic bath (model M1800H-E, Bransonic, Danbury, CT, USA), GO/SWCNTs-COOH dispersions were filtered in a vacuum (−0.04 bar, pressure) through PTFE disks (diameter: 47 mm, average pore size: 0.5 µm, Durapore©, Merck KGaA, Darmstadt, Germany). At the end of filtration, after drying at room temperature, GO-BP membranes were peeled from PTFE disks (average D: 35 ± 2 mm, t: 65 ± 1 µm).

### 2.3. Thermodynamics and Kinetics of Dyes Adsorption

Adsorption experiments were conducted by placing one GO-BP disk (50 mg) in 400 mL of dye aqueous solutions at different initial concentrations (10, 25, 50, and 100 mg L^−1^) for each pollutant. All experiments were performed under stirring (180 rpm) by using an orbital shaker (PSU-10i, Biosan, Italy) and adsorption was evaluated for 48 h at pH = 6 and at the following temperature values: 298 and 313 K. The amount of adsorbed dye onto GO-BP membranes is calculated in terms of removal efficiency (RE%) by using Equation (1):

(1) $$RE\%=\frac{\left(C_{0}-C_{e}\right)\cdot 100}{C_{0}}$$

where $C_{0}$ and $C_{e}$ represent the initial and at equilibrium concentrations, respectively.

Langmuir and Freundlich isotherm models were adopted to fit adsorption isotherms at 298 K and 313 K [27,28]. According to Langmuir model, it is assumed that there is a one-to-one correspondence between active sites on adsorbent surface and adsorbate molecules; therefore, adsorption follows a monolayer distribution which can be described by the following Equation (2):

(2) $$q_{e}=\frac{q_{m}K_{L}C_{e}}{1+K_{L}C_{e}}$$

with $q_{e}$, $q_{m}$ and $K_{L}$ being, respectively, the maximum adsorption experimental capacity (mg g^−1^), the maximum adsorption capacity estimated by the Langmuir model (mg g^−1^), and the ratio between adsorption and desorption rate (L mg^−1^). Flipping the terms of Equation (2) and rearranging them, the linearized form of the Langmuir model becomes

(3) $$\frac{1}{q_{e}}=\left(\frac{1}{K_{L}q_{m}}\right)\frac{1}{C_{e}}+\frac{1}{q_{m}}$$

where $\frac{1}{q_{e}}$ is directly proportional to $\frac{1}{C_{e}}$. The other common isotherm model, represented by Freundlich, instead assumes that adsorption onto a heterogeneous surface occurs with uniform energy, with no restrictions to the formation of a single layer. In this case, the model is represented by the following Equation (4):

(4) $$q_{e}=K_{F}C_{e}^{\frac{1}{n_{F}}}$$

with $K_{F}$ (L mg^−1^) and $n_{F}$ being the constants of the model, whose values depend on temperature. The linearized form of Freundlich model becomes

(5) $$\log q_{e}=\log K_{F}+\frac{1}{n_{F}}\log C_{e}$$

where by plotting $\log q_{e}$ vs. $\log C_{e}$, a straight line can be obtained, whose slope is $\frac{1}{n_{F}}$. According to thermodynamics laws, the standard change in free Gibbs energy ($∆G^{{}^{\circ}}$) is calculated as follows:

(6) $$∆G^{{}^{\circ}}=-RT\ln K_{eq}$$

with $K_{eq}$ being the equilibrium constant, derived from the adopted isotherm model ($K_{L}$) and converted from the dimensional to the dimensionless through Equation (7) [29]:

(7) $$K_{eq}=K\cdot 10^{3}\cdot {MM}_{ads}$$

where ${MM}_{ads}$ (g mol^−1^) is the molar weight of adsorbate. Considering that

(8) $$∆G^{{}^{\circ}}=∆H^{{}^{\circ}}-T∆S^{{}^{\circ}}$$

substituting Equation (6) into Equation (8) and rearranging it, Equation (9) is obtained as follows:

(9) $$\ln K_{eq}=-\frac{∆H^{{}^{\circ}}}{R}\frac{1}{T}+\frac{∆S^{{}^{\circ}}}{R}$$

which is the linearized form of Equation (8) from which $∆H^{{}^{\circ}}$ and $∆S^{{}^{\circ}}$ can be calculated, assuming that $∆H^{{}^{\circ}}$ and $∆S^{{}^{\circ}}$ are independent of temperature in the range investigated (298–313 K) [29].

Dye adsorption curves were also fitted with a pseudo-first-order (PFO) and a pseudo-second-order (PSO) model in order to derive adsorption kinetic constants. According to the Lagergren first-order kinetic model, adsorption process can be described as follows [30]:

(10) $$\frac{dq_{t}}{dt}=k_{1}\left(q_{e}-q_{t}\right)$$

with $q_{t}$ and $q_{e}$ being, respectively, dye adsorption capacity per unit of adsorbent mass (mg·g^−1^) at time *t* and at equilibrium (*e*), while $k_{1}$ is the Lagergren adsorption rate constant (min^−1^). After integration, the linearized form of Equation (10) becomes Equation (11):

(11) $$\ln\left(q_{e}-q_{t}\right)=\ln q_{e}-k_{1}t$$

if a second-order model is adopted, adsorption process is best described by Equation (12) [31]:

(12) $$\frac{{dq}_{t}}{dt}=k_{2}{\left(q_{e}-q_{t}\right)}^{2}$$

with $k_{2}$ (g·mg^−1^·min^−1^) being the kinetic pseudo-second-order rate constant. After integration, Equation (12) could be rearranged in the following liner form:

(13) $$\frac{1}{q_{e}-q_{t}}=\frac{1}{q_{e}}+k_{2}t$$

### 2.4. Characterizations

Scanning electron microscopy (Leica LEO 420, Leica Microsystems, Cambridge, UK) was used to elucidate the morphology of GO-BP membranes. Samples were covered with an ultrathin Au layer (10 nm) before investigation by using Auto Sputter Coater (Agar, Cambridge, UK), and analysis was performed under the following conditions: accelerating voltage of 10 kV, vacuum conditions of 1·10^−6^ Torr. A Nanoscope III (Digital Instruments, Santa Barbara, CA, USA) was used for AFM measurements. Mechanical strength of GO-BP membranes was determined with a Sauter TVO-S tensile tester, equipped with a Sauter FH-1k digital dynamometer and AFH LD software 2.0.2.1 (Sauter GmbH, Balingen, Germany). Rectangular strips (*l × w* = 3 cm × 5 mm) were obtained from GO-BP membranes tested for elongation measurements at a strain rate of 0.1 mm·min^−1^. Thermogravimetric analysis of GO-BPs was performed on a TGA—STA 2500 Regulus simultaneous thermal analyzer (Netzsch, Selb, Germany) using a temperature schedule defined as follows: T ramp: 20–750 °C; heating rate: 5 °C·min^−1^; flowing gas: Ar:O_2_ mixture (99:1); flow rate: 100 sccm. Contact angle measurements were performed at 25 °C under static conditions by using a goniometer (Nordtest, Serravalle Scrivia AL, Italy). A drop (2 µL) of water was deposited on a sample surface and contact angle values were obtained through averaging the set tangents measurements on both droplet visible edges picked up on five different positions of each sample. GO-BPs porosity, *p*, was assessed through the gravimetric method at 25 °C. Membranes were dipped into a wetting liquid (3M-FC-40, 3M Italia Srl, Pioltello, Milan, Italy) in order to measure their weight difference before and after immersion. Then, porosity is calculated through the following equation

(14) $$P=\frac{\frac{m_{w}-m_{d}}{d_{w}}}{\frac{m_{w}-m_{d}}{d_{w}}+\frac{m_{d}}{d_{m}}}$$

where it is assumed that $m_{d}$ and $m_{w}$ are, respectively, the weight of dry samples and that of wet samples, $d_{w}$ is the density of wetting liquid (1.855 g·cm^−3^), and $d_{m}$ is the average BP density (0.60 ± 0.03 g·cm^−3^, which was previously determined through measurements of buckypaper surface area, weight, and thickness). A zeta potential analyzer (SurPASS TM 3, Anton Paar Italia S.R.L., Turin, Italy, equipped with an adjustable gap cell) was employed to measure the surface charge of GO-BPs as a function of pH value (pH range = 3–9, T = 25 °C). After being mounted on sample holders, each membrane (cross section: 2 × 1 cm^2^) was subjected in pairs to changes in pH by adding 0.05 M of HCl. Z potential was finally calculated from streaming potential measurements through Helmholtz and Smoluchowski equation [32]. FT-IR spectra on pristine SWCNTs-COOH and GO powders, as well as on GO-BP membranes, were recorded on a Fourier transform infrared (FT-IR) spectrometer (Bruker ALPHA FT-IR, Milan, Italy) equipped with a A241/D reflection module. The spectra were collected in the 375−4000 cm^−1^ region and OPUS Bruker software 7.8 was used for their interpretation. Dye concentration was determined through absorbance measurements on an Evolution 201 spectrophotometer (ThermoFisher Scientific, Hillsboro, OR, USA), equipped with 1.0 cm quartz cells, by using calibration curves of investigated pollutants. Absorbance values were collected in correspondence with λ_max_ of each pollutant, being 602 nm for AB29, 590 nm for CV, 518 nm for EY, 620 nm for MG, and 542 nm for RB.

## 3. Results

### 3.1. GO-BPs Characterization

**Figure 1:**
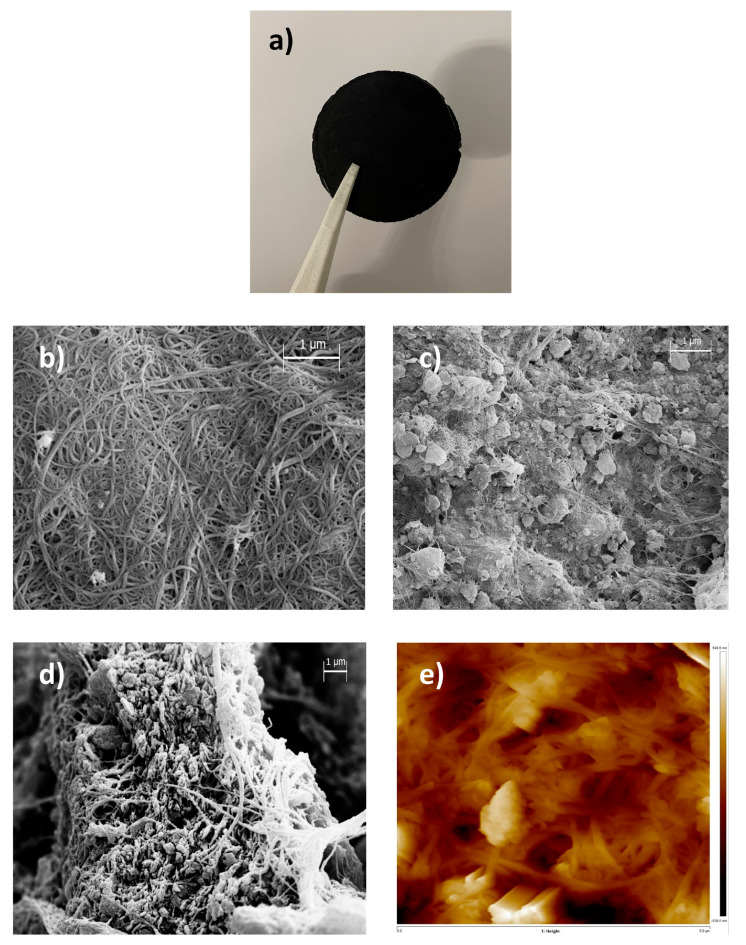
GO-BP membrane peeled from the PTFE filter (**a**). SEM images of (**b**) neat BP surface, (**c**) GO-BP surface, and (**d**) GO-BP cross section. AFM image of GO-BP surface (**e**).

**Figure 2:**
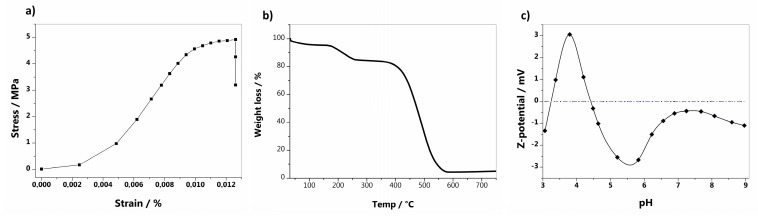
GO-BPs: stress vs. strain curve (**a**), TGA (**b**), and Z potential (**c**).

**Figure 3:**
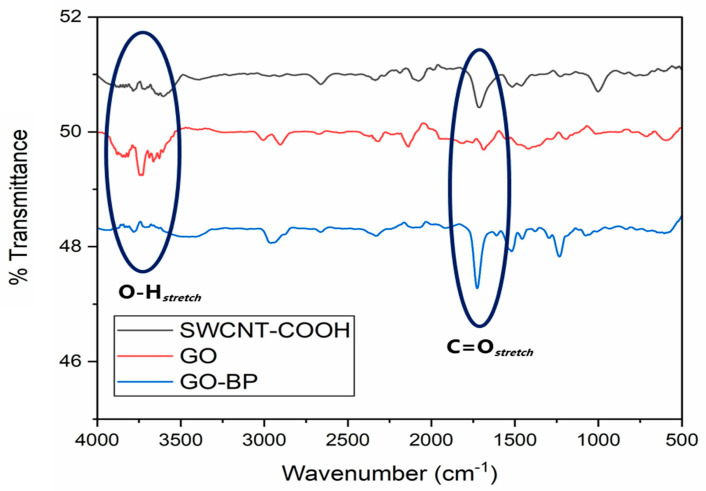
FT-IR spectra of SWCNTs-COOH (black), GO (red), and GO-BPs (blue).

GO-BP membranes were prepared by vacuum-assisted filtration of the GO/SWCNTs-COOH initial mixture. The amount of GO to be incorporated in the neat BP membrane has been previously studied by the authors of [26], who found that the optimal ratio GO:SWCNTs-COOH is 75:25 *w*/*w*%. This choice provides the maximum amount of graphene oxide in the membrane, which is highly adsorptive for dyes due to its planar structure, and, at the same time, the preservation of BP standing-alone properties, facilitating its removal at the end of the adsorption process (Figure 1a). Furthermore, by adopting a technique such as the wet method, together with the ease of operation, great advantages come from the use of low amounts of an eco-friendly solvent (methanol) and from the absence of post-synthetic treatments. Despite the huge concentration of graphene oxide, GO-BP membranes maintain the typical texture of BPs (Figure 1b), where CNTs filaments are braided between themselves, due to π−π and van der Waals interactions, forming an entangled net. Along the outer surface of CNTs bundles, GO sheets are positioned, where π−π and van der Waals interactions between the flat surface of GO and the round one of CNTs keep GO flakes incorporated and homogeneously disposed within the membrane (Figure 1c,d). This arrangement of alternance among CNT bundles and GO flakes is also noticeable by AFM analysis conducted on GO-BPs (Figure 1e), confirming the texture already observed through scanning electron microscopy: a layer of tangled filaments marked all across the surface by a huge presence of GO flakes. Surface roughness measurements onto GO-BPs membranes were evidenced by the following values: R_a_ = 101 ± 15 nm, R_q_ = 135 ± 19 nm, and R_max_ = 1019 ± 150 nm, by which it could be immediately noticed that surface roughness impacts GO-BPs’ thickness by a maximum of 1.5%. Thanks to the high number of interactions between GO and CNTs, GO-BPs exhibit a remarkable mechanical stability, as confirmed by the tensile strength measurements performed on them. Young modulus of as-obtained membranes is found to be 0.7 ± 0.1 GPa, with a tensile strength value of ~5 MPa (Figure 2a). It is worth noting that the inclusion of graphene oxide into a BP membrane inevitably changes the mechanical properties of pure BPs. The latter are entirely made of carbon nanotubes, which can support and strengthen the membrane network through their long tubular structure. In contrast, in GO-BPs, 75% of CNTs are completely replaced by graphene oxide, which cannot cover the role of missing CNTs with its flat and planar morphology. Therefore, as expected, the mechanical stability of GO-BPs is slightly lower than neat BPs, whose Young modulus is typically around 0.9 GPa. On a larger scale, based on materials outside of buckypapers, GO-BP tensile strength is still high, ensuring great mechanical stability for the purpose of this work. The presence of GO is additionally notified in the TGA curve of GO-BP membranes, whose final shape results are affected by thermal behavior of two component materials (Figure 2b). As evidence of this, TGA thermogram modifies the typical shape observed in neat BPs, in which weight loss shows a single drop at around 450 °C that confirms BPs’ thermal stability at this temperature [21], into a ladder pattern characterized by two fundamental steps: the first one, at around 200 °C, which can be entirely ascribed to GO, due to the removal of oxygen functional groups of which GO is loaded [33], and the second one, at around 400 °C, coming from the oxidative pyrolysis of carbon framework of CNTs and GO. Porosity measurements on GO-BPs show a porosity percentage of 61 ± 5%, pointing out a decrease in this value when compared to that of neat BPs (74 ± 5%), as reported in a previous work [26]. This reduction in porosity could be ascribed to the combination of multiple factors, including the high amount of GO used to prepare the membrane together with a closer packing of GO sheets. Due to their chemical composition, made of carboxylic and hydroxylic groups, it is assumed that GO-doped buckypapers are hydrophilic. Contact angle measurements clearly confirm this characteristic, with a CA value equal to 40.7° ± 0.5°. GO-BP surface charge, in terms of Z potential, as a function of pH is reported in Figure 2c. As shown in the figure, the point of zero charge (pH_ZCP_), i.e., the pH value at which net surface charge is 0, is 4.4. Consequently, when pH < pH_ZCP_, GO-BP surface is positive with -COOH and -OH groups in their acid form; otherwise, if pH > pH_ZCP_, carboxylic and hydroxylic groups start deprotonation and GO-BP surface becomes more negative with increasing pH. Figure 3 finally reports the FT-IR spectra registered on SWCNTs-COOH, GO, and GO-BP membrane. The most relevant peaks in these spectra are essentially confined in the region at around 1750 cm^−1^, where stretching of C=O occurs, and at around 3500 cm^−1^, due to O-H stretching, confirming the presence of carboxylic functionalization on both components and on GO-BPs. Therefore, in consideration of all morphological, thermal, and chemical evidence coming from SEM, AFM, TGA, FT-IR, while also including tensile strength, porosity, and CA measurements, it can be concluded that GO doping of BPs has been successfully achieved.

### 3.2. Dyes Adsorption

GO-BP membranes were projected for dye adsorption from aqueous solutions. To investigate their adsorptive properties, five different pollutants were chosen, according to reasons related to natural chemical composition, in order to cover different classes of dyes and wastewater abundance. Based on this premise, the following dyes were selected: Acid Blue 29 (AB29, an anionic disazo dye), Crystal Violet (CV, a triarylmethane dye), Eosyn Y (EY, a triarylmethane dye), Malachite Green (MG, a triarylmethane dye), and Rhodamine B (RB, a xanthene dye). The incorporation of graphene oxide into BP membranes is functional to the enhancement of BP capture properties without renouncing their self-standing ability. Literature works clearly show that high recovery percentages can be obtained when graphene oxide is employed as an adsorbent [34,35]. This can be ascribed to the extraordinary compatibility between GO flat morphology and chemical planar structure of the majority of dyes, which allows the maximization of the number of interactions between the two parties involved, thus favoring their adsorption. Furthermore, the option to toggle the charge on GO surface, alternating between a neutral or charged form depending on the pH, reinforces dyes adsorption through the additional establishment of electrostatic interactions, with a lot of dyes being cationic or anionic in chemical nature [36,37,38]. The employment of GO in the form of powder brings some difficulties during the adsorbent recovery phase, due to its nanosized dimensions. As a result, to overcome this problem in the present work, GO has been incorporated in buckypaper membranes, preparing an alternative BP version that aims to combine high adsorption properties of GO, enhancing those already held by pristine BPs, with their mechanical stability and self-standing ability.

**Figure 4:**
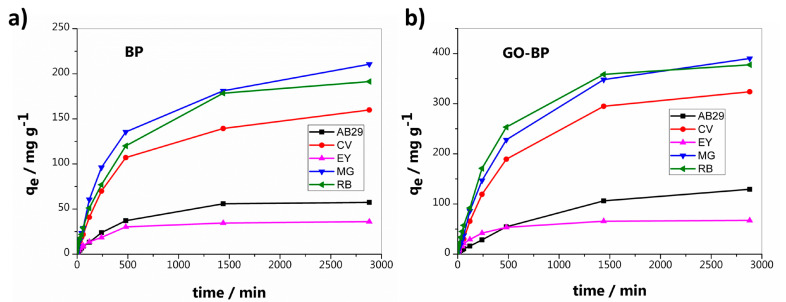
Adsorption curves of AB29, CV, EY, MG, and RB of (**a**) neat BPs and (**b**) GO-BPs.

**Figure 5:**
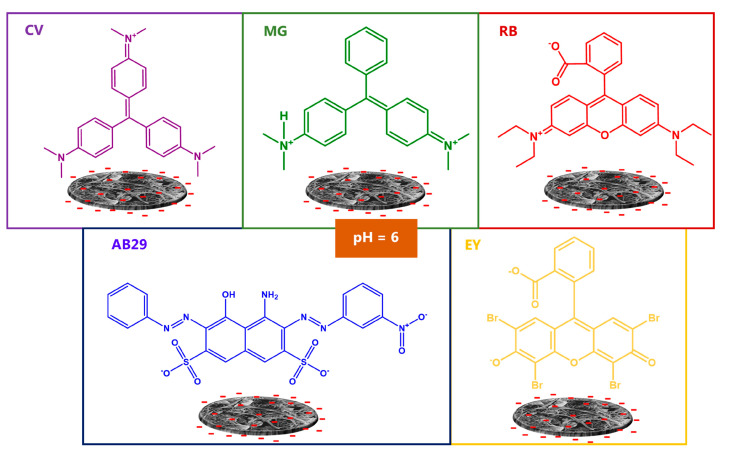
Schematic representation of electrostatic interactions established between GO-BPs and dye molecules.

Adsorption experiments were performed in batches, at room temperature, and at pH = 6 in order to simulate a condition similar as much as possible to that of dyes in wastewater. One GO-BP disk was employed in 400 mL of pollutant solution, previously prepared at an initial concentration value of 50 mg L^−1^. Capture properties of GO-BP were evaluated for a period of 48 h by taking samples at cadenced intervals of 5 min and monitoring the decrease in dye concentration through UV-Vis spectroscopy, set at λ_max_ for each pollutant. Figure 4a reports the adsorption of AB29, CV, EY, MG, and RB by GO-BPs after a period of 48 h. Adsorption curves clearly indicate that, to parity of initial concentration (50 mg L^−1^), GO-BP capture properties differ enough among dyes, with RB and MG showing the highest values of recovery (RE_RB_ = 94.5%, RE_MG_ = 95.5%). In fact, comparing curves of Figure 4a, it is revealed that, after 8 h, CV, MG, and RB have already halved their initial concentration, attaining values of 27.1, 22.6 and 18.3 mg L^−1^, respectively, while EY and AB29 remain a long way off from 25 ppm, moving instead towards the equilibrium values. Furthermore, concentrations of CV, MG, and RB drop quite abruptly at an ulterior 30% in the remaining time before 24 h, after which adsorption equilibrium is reached. In fact, after 24 h, only a slight increase in RE% (~5%) is observed up to 48 h except for EY, which instead achieves equilibrium at *t* = 240 min. These differences in adsorption behavior of dyes are ascribable to chemical interactions established between GO-BP membrane and the investigated pollutant. Typical interactions involved during adsorption include π−π, Van der Waals, and electrostatic interactions. At pH = 6, at which experiments are performed, GO-BP surface is negatively charged. On the other hand, depending on their pK_a_ values, dyes molecules could exhibit a neutral or charged form. In particular, at pH = 6, CV (pK_a1_ ≈ 1.15 and pK_a2_ ≈ 1.8), rapidly losing two protons under acidic conditions, maintains a positive charge for one in three nitrogens in its chemical structure while MG (pK_a_ ≈ 6.9) is doubly protonated for its N atoms. RB (pK_a_ ≈ 4.8), due to the presence of a carboxylate group, instead exhibits a neutral form. Finally, EY (pK_a_ ≈ 4.8) and AB29, in consequence of the presence on their structures of a carboxylate and two sulfonic groups, respectively, are both negatively charged. Based on these premises, electrostatic interactions play a pivotal role in the adsorption of investigated dyes on GO-BP membranes (Figure 5) [34,39]. In fact, as shown in Figure 4a, the lowest adsorption values are observed for EY and AB29, where adsorption phenomenon, torn between two conflicting forces, suffers prevalently from electrostatic repulsion between dye and membrane surface (both negatively charged), despite the positive tendency to recovery due to π−π and Van der Waals interactions. Contrariwise, in the case of CV, MB, and RB, both forces, electrostatic and hydrophobic, point in the same direction since aromatic structure of investigated dyes suits perfectly with the flat morphology of GO-BPs, and adsorption is strongly assisted by electrostatic attraction among positive dyes and negative charge on the membrane surface. Furthermore, the highest charged quantity (MG) and the highest recovery value are observed, suggesting the importance of electrostatic interactions in this process. This behavior, leading to a preferential adsorption of cationic dyes on GO surface owed to electrostatic attraction, has been already evidenced by Ramesha et al. [39]. The authors indeed reported the difference in GO adsorption performance towards cationic and anionic dyes, concluding that the former were better retained on graphene oxide thanks to the primary role covered by charge-based interactions. To better support the importance of GO in dye adsorption, experiments were also conducted on neat BPs, maintaining the same operational conditions: initial concentration value of 50 mg L^−1^, solution volume of 400 mL, pH of 6 and room temperature. As clearly evidenced in Figure 4b, where BPs’ adsorption values against dyes are reported, GO-BPs’ membranes configure themselves as better adsorbents for dye capture from water, doubling the amount of contaminants retained on their surface for all five investigated dyes. Indeed, neat BPs follow the behavior already outlined by GO-BPs, presenting the best adsorption values for MG and RB. However, the lack of a major number of -COOH groups (3–4% functionalization of SWCNT-COOH vs. 8–10% of GO), which electrostatic interactions depend on, together with the absence of a proper chemical structure able to match perfectly with dyes planar shape, represent the major obstacle to dyes full capture, making BPs surely good adsorbents but susceptible to great improvement with the addition of the correct dopant agent, i.e., graphene oxide. For the reasons listed above, and due to their lower performance, neat BPs have been excluded from further adsorption experiments, for which GO-BPs appear to be the only ideal candidate.

Taking into consideration the adsorption capacity values of GO-BP membranes at 50 mg L^−1^, the following trend can be derived: MG (390.17 mg g^−1^) ≈ RB > CV > AB29 > EY (67.09 mg g^−1^), with all *q_max_* values reported in Table 1. Further evidence of this trend comes from the calculation of dye distribution coefficient in Equation (15), K_D_, on GO-BP membrane [40]:

(15) $$K_{D}=\frac{{\left[dye\right]}_{GO-BP}}{{\left[dye\right]}_{sol}}$$

with ${\left[dye\right]}_{GO-BP}$ (mg Kg^−1^) and ${\left[dye\right]}_{sol}$ (mg L^−1^) being the equilibrium dye concentrations adsorbed on GO-BP membrane and in the solution, respectively. Generally, when $\log K_{D}>5$, high affinity between adsorbent and adsorbate substists. *LogK_D_* values for AB29, EY, CV, MG, and RB are reported in Table 1. As expected, high affinity is observed when the pollutants are MG and RB (logK_MG_ = 5.23 and logK_RB_ = 5.14), confirming the greatest percentages of adsorption recovery on GO-BP membranes, followed by CV (logK_CV_ = 4.50), AB29 (logK_AB29_ = 3.58), and EY (logK_EY_ = 3.21).

In the analysis of adsorption curves, both kinetic models (PFO and PSO) have been used for fitting. Lagergren adsorption rate constants, as well as pseudo-second-order rate constants, each followed by their corresponding R^2^ values, are reported in Table 1. As corroborated by R^2^ values, a first-order fitting suits properly the behavior observed experimentally, confirming that the adsorption process is faster for MG (*k*_1_ = 0.00156) and RB (*k*_1_ = 0.00207), which also benefit from the highest adsorption values. Interestingly, despite its low recovery percentage, EY exhibits a kinetic constant quite similar to RB (*k*_1_ = 0.00259 vs. *k*_1_ = 0.00207, respectively), indicating that EY adsorption process on GO-BP membranes is quick, so much so that equilibrium is achieved after only 240 min, while a five-fold time is required for the other dyes to reach the plateau region. This potential high value of EY kinetic constant should not mislead with the GO-BP adsorption capacity for this dye. The latter indeed depends on the typology and quality of interactions between EY and GO-BP surface. On the other hand, the kinetic model studies the process in terms of rate of occupied sites. Therefore, this apparent contradiction in-between kinetic constant and adsorption capacity values simply suggests that EY rapidly occupies adsorbent sites available on GO-BP surface until hydrophobic interactions prevail. Afterwards, due to their negative charges, the increasing amount of pollutants on the GO-BP membrane exposes both involved parts of a notably electrostatic repulsion, which becomes predominant and interrupts the addition of further pollutants on GO-BP surface.

In order to evaluate maximum adsorption capacity of GO-BP membranes, adsorption experiments were repeated at increased dye concentrations of 100 mg L^−1^ each. Final values are reported in Table 2, attesting the high potentiality of GO-BP membranes towards RB (467 mg g^−1^) and MG (493 mg g^−1^), for which *q_max_* rises by a further 25% when initial concentration moves from 50 to 100 mg L^−1^.

For the construction of adsorption isotherms, experiments for all selected dyes were also performed at 10 and 25 mg L^−1^, testing each concentration at two different temperature values: 298 K and 313 K. Langmuir and Freundlich models were both used to fit experimental results (Figures S1 and S2). As shown by R^2^ values contained in Table 3, where all discussion parameters are reported, adsorption isotherms for all investigated dye results best described by the Langmuir model, confirming the trend that has already been found: MG and RB are the best retained pollutants, exhibiting the highest values of K_L_ (K_MG_ = 0.731 and K_RB_ = 0.576) and thus the major affinity with GO-BPs, followed by CV (K_CV_ = 0.140), AB29 (K_AB29_ = 0.034), and EY (K_EY_ = 0.009). The increment experienced by K_L_ at 313 K, common to all investigated dyes, suggests that adsorption on GO-BPs is favored by higher temperatures, with GO-BPs’ affinity towards dyes maintaining the same preference order observed at 298 K (MG ≈ RB > CV > AB29 > EY). This high adsorption potential of GO-BPs also found its reason from the close correspondence between the two values of *q_max_*, which are experimentally found and theoretically calculated by extrapolation from the slope of the linearized form of Langmuir fitting, demonstrating that all potential active sites on membrane surface are occupied or almost occupied, and are thus prone to saturation, especially when adsorption is performed at higher temperatures. In this sense, the only exception is represented by EY, exhibiting the major discrepancy from the theoretical value of *q_max_*. This misplaced note simply points out that even if GO-BPs possess active sites in which EY can be hosted, most of them are unavailable because of factors related to adsorption conditions, such as pH. In fact, at pH = 6, charge distribution on EY is counterproductive, since EY suffers from electrostatic repulsion with GO-BP negative surface. Therefore, in this scenario, adsorption is hampered and, excluding the contribution coming from the lower power of hydrophobic forces, many potential active sites on GO-BPs remain empty. The other important factor in support of GO-BPs’ high adsorption capacity is related to the thermodynamic character of the process, favorable or not, that is normally dependent on the value of n_F._ According to Freundlich modeling, adsorption is favorable when 0 < 1/n_F_ < 1. By linear fitting, it emerges that the adsorption process is favorable in all the five cases, with a marked aptitude for capturing for MG, RB, and CV, where 1/n_F_ values come close to zero. Thermodynamic parameters (ΔG°, ΔH° and ΔS°), calculated from Equations (6) and (9), are reported in Table 4. Corroborating the thesis on the value of n_F_, it is found that ΔG° is negative for all selected dyes, demonstrating that the adsorption process is spontaneous and thus favorable. In compliance with the preference degree of affinity, ΔG° is more negative for MG, RB, and CV, supporting the fact that these dyes are those mostly retained from GO-BP membranes. Furthermore, at 313 K, as expected from higher values of K_L_, ΔG° negatively increases, validating the spontaneity of the adsorption process even at high temperatures, where the same scale order observed at room temperature (ΔG°_MG_ ≈ΔG°_RB_ < ΔG°_CV_ < ΔG°_AB29_ < ΔG°_EY_) is respected. Being favored by temperature increase, it is assumed that adsorption be an endothermic process. ΔH° calculation results in a positive value for all dyes, confirming the above-mentioned statement.

**Figure 6:**
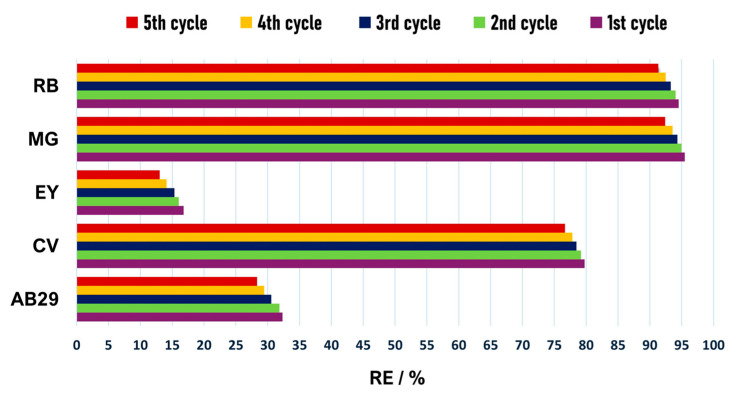
Recovery percentages of GO-BPs towards AB29, CV, EY, MG, and RB in 5 regeneration cycles (C_AB29_ = C_CV_ = C_EY_ = C_MG_ = C_RB_ = 50 mg L^−1^).

Considering stability and efficiency over long-term use, GO-BP membranes were recycled and reused in successive adsorption tests, repeating experiments for each dye at 50 mg L^−1^ for five times. Regeneration step was performed by washing GO-BP membranes at the end of each adsorption cycle with an ethanol:water (2:1) solution for three times, and then drying GO-BPs at room temperature. With a discrepancy in RE% values of 4% at maximum between the first and last cycle, recycle experiments ascertained the reusability of GO-BP membranes for subsequent adsorption tests, maintaining their recovery percentages (Figure 6) towards dyes up to five times from the first experiment.

### 3.3. Comparison with Other Adsorbents

To better comprehend the adsorption potential of GO-BP membranes towards dyes, a comparison of some literature data is proposed in Table 2. The table emphasizes the most relevant aspects to be considered when designing the adsorption process, such as pH, amount of adsorbent, and initial dye concentration, while also providing the q_max_ value associated with each cited work. Despite the authors’ intent to narrow the field to only references concerning the employment of GO, the scarcity or, in some cases, the lack of literature data about the adsorption of investigated dyes through GO forced them to expand the research and consider more adsorbents than only graphene oxide. Considering the data presented in Table 2, it is evident that, where the comparison is possible, GO has an additional value than some natural adsorbents, such kaolin, or rice husk, suggesting how fundamental the contribution of GO large surface area is to dyes adsorption. This is particularly evident in the case of AB29, where the use of GO-BP membranes enables the achievement of a maximum adsorption capacity value three/four times higher than those reported in the literature using a total adsorbent amount significantly lower (50 mg vs. 300/500 mg). A similar trend is observed for EY, where GO-BP membranes ensure a better adsorption capacity than some conventional adsorbents (zeolite Y). However, it is clearly evident that the difference is the pH. Under acidic conditions, EY exhibits a less negative form that can electrostatically interact with GO-positive surfaces, thus favoring adsorption. This can explain the high q_max_ values (217 and 555 mg g^−1^, respectively) joined with GO by Veerakumar et al. [49] and Ahmad et al. [50], exceeding adsorption capacity results found in this work, where experiments were performed at pH = 6. With their strong interactions with GO surface, CV, MB, and RB are the favored substrates to be captured at pH = 6. Confirming the behavior already observed for AB29, GO-BP membranes are able to efficiently retain large quantities of the above-mentioned dyes, exhibiting q_max_ values, at constant adsorbent amounts similar, or even greater, than those reported in the literature. The possibility to entrap huge amounts of dyes by using low-adsorbent contents marks the great potential of GO-BP membranes developed in this work as excellent adsorbents in dye recovery.

## 4. Conclusions

In this work, an alternative version of CNT buckypapers, based on GO incorporation into the inner structure of BPs, is proposed. Physico-chemical characterization (SEM, AFM, TGA, FT-IR, CA, and Young modulus measurements) of the as-obtained system (GO-BPs) confirmed the successful doping of BPs with graphene oxide (75 *w*/*w*%), preparing a membrane that takes advantage of both components’ properties, such a large surface area and huge adsorption capacity (typical of GO) with high mechanical and chemical stability, standing-alone property, and flexibility (specifics of BPs). The preparation of GO-BP membranes has the aim of proposing GO-BPs as adsorbents in dye removal from aqueous solutions. The authors selected five dyes (AB29, CV, EY, MG, and RB), differing for chemical structure and classification, and tested GO-BPs’ adsorption performance starting from C_i_ = 50 mg L^−1^ per dye (pH = 6 and r. t.). Results pointed out that the best adsorption performance are achieved when dye capture on GO-BP surface is promoted not only by hydrophobic interactions but also by electrostatic ones, the latter contributing dramatically to enhancing the amount of retained dye. Adsorption isotherms provide evidence that dye capture onto GO-BP membranes is a favorable, endothermic, and spontaneous process, with ΔG° becoming more negative at higher temperature values (313 K). According to derived thermodynamic values, the degree of affinity of GO-BPs towards investigated dyes could be summarized as follows: MG ≈ RB > CV > AB29 > EY. Kinetic studies also confirmed the predominance of MG, RB, and CV in being the preferable adsorbates to be captured, exhibiting a fast adsorption process (*k_MG_*, *k_RB_* ≈ 2*k_AB29_*). In view of this, maximum adsorption capacities have been calculated, reporting the following values: MG (493.44 mg g^−1^), RB (467.35 mg g^−1^), CV (374.53 mg g^−1^), AB29 (162.65 mg g^−1^), and EY (85.38 mg g^−1^).

## Figures and Tables

**Table 1:** *K_D_*, *q_max_*, and kinetic constant *k*_1_ and *k*_2_ values for AB29, CV, EY, MG, and RB at C_dye_ = 50 mg L^−1^, pH = 6, r.t.

Dye	LogK_D_	Maximun Adsorption Capacity (mg g^−1^)	1st Order Kinetic Constant k_1_ × 10^4^ (min^−1^)	R^2^	2nd Order Kinetic Constant k_2_ × 10^6^ (g mg^−1^ min^−1^)	R^2^
**AB29**	3.58	129.31	11.4 ± 0.2	0.9964	22.0 ± 1.0	0.9784
**CV**	4.50	323.74	16.9 ± 0.2	0.9988	21.3 ± 1.4	0.9532
**EY**	3.21	67.09	25.9 ± 1.0	0.9845	427.5 ± 35.3	0.9296
**MG**	5.23	390.17	15.6 ± 0.3	0.9968	14.3 ± 0.8	0.9672
**RB**	5.14	377.53	20.7 ± 0.3	0.9983	33.3 ± 2.5	0.9383

**Table 2:** Comparison of maximum adsorption capacities of different adsorbents towards AB29, CV, EY, MG, and RB at room temperature.

Dye	Adsorbent Typology	Adsorbent Dose/(mg)	pH	C_dye_/ (mg L^−1^)	q_max_/ (mg g^−1^)	Ref.
**AB29**	MgO-SiO_2_	500	10	100	44.9	[41]
	K_2_CO_3_-activated olive pomace boiler ash	300	6	100	38.48	[42]
	GO-BP	50	6	100	162.65	This work
**CV**	Kaolin	100	7	100	45.0	[43]
	NaOH-modified rice husk	1000	7	50	44.87	[44]
	MWCNT-COOH	10	6	500	100.0	[45]
	GO	25	6	200	487.80	[46]
	GO-BP	50	6	100	374.53	This work
**EY**	Zeolite Y	100	2.5	60	52.91	[47]
	CNTs-incorporated eucalyptus	20	6	50	49.15	[48]
	GO	300	1	50	217.33	[49]
	GO/ZnO nanocomposite	230	2	100	555.55	[50]
	GO-BP	50	6	100	85.38	This work
**MG**	Fe_3_O_4_-AC	100	6	300	217.68	[51]
	GO/aminated lignin aerogels	20	8	50	113.5	[52]
	GO-Cellulose-Cu	1000	7	400	207.1	[53]
	MWCNT-COOH	40	7	100	142.85	[54]
	rGO	50	7	200	476.2	[55]
	GO-BP	50	6	100	493.44	This work
**RB**	Magnesium silicate/carbon composite	100	6.5	600	244	[56]
	Montmorillonite/GO	300	7	150	625.0	[57]
	Benzene carboxylic acid/GO-zeolite	10	3	500	67.56	[58]
	MWCNT-COOH	50	7	100	42.68	[59]
	GO-BP	50	6	100	467.35	This work

**Table 3:** Langmuir and Freundlich parameters determined from AB29, CV, EY, MG, and RB adsorption isotherms.

			Langmuir			Freundlich		
	T (K)	q_exp_ (mg/g)	q_max_ (mg/g)	K_L_ (L/mg)	R^2^	K_F_ (L/mg)	n_F_	R^2^
**AB29**	298	162.6	227.3	0.034	0.9982	14.7	1.7	0.9660
	313	177.3	222.2	0.051	0.9986	21.5	1.9	0.9606
**CV**	298	374.5	476.2	0.140	0.9961	81.0	2.3	0.8543
	313	407.6	454.5	0.338	0.9984	120.2	2.7	0.8468
**EY**	298	85.4	200.0	0.009	0.9979	3.8	1.4	0.9678
	313	101.5	227.3	0.012	0.9975	5.0	1.4	0.9629
**MG**	298	493.4	526.3	0.731	0.9941	180.7	3.0	0.7881
	313	504.1	500.0	1.667	0.9960	222.3	3.5	0.7764
**RB**	298	467.3	526.3	0.576	0.9964	165.3	2.9	0.8224
	313	478.1	500.0	1.428	0.9973	207.9	3.4	0.7750

**Table 4:** Thermodynamic parameters of AB29, CV, EY, MG, and RB adsorptions on GO-BPs.

	T (K)	ΔG (kJ/mol)	ΔH (kJ/mol)	ΔS (J/(K·mol))
**AB29**	298	−24.5	20.4	150.5
	313	−26.7	20.4	150.5
**CV**	298	−26.9	45.6	243.4
	313	−30.6	45.6	243.4
**EY**	298	−21.4	16.8	128.3
	313	−23.3	16.8	128.3
**MG**	298	−30.7	42.6	246.1
	313	−34.4	42.6	246.1
**RB**	298	−30.8	47.0	261.2
	313	−34.8	47.0	261.2

## Data Availability

The original contributions presented in this study are included in the article/Supplementary Material. Further inquiries can be directed to the corresponding author.

## Supplementary Materials

The following supporting information can be downloaded at: https://www.mdpi.com/article/10.3390/nano15110782/s1, Figure S1. Adsorption isotherms at 298 K for (a) AB29, (b) CV, (c) EY, (d) MG, (e) RB, according to Langmuir and Freundlich models. Figure S2. Adsorption isotherms at 313 K for (a) AB29, (b) CV, (c) EY, (d) MG, (e) RB, according to Langmuir and Freundlich models.

## Abbreviations

The following abbreviations are used in this manuscript:

SWCNT-BP	single-walled carbon nanotube buckypaper
MWCNTs	multi-walled carbon nanotubes
GO	graphene oxide
BP	buckypaper
AB29	acid blue 29
CV	crystal violet
EY	eosyn Y
MG	malachite green
RB	rhodamine B
