# Supplementary material for: Graphene Oxide-Doped CNT Membrane for Dye Adsorption
Source: Nanomaterials (Basel). 2025 May 22;15(11):782. doi: 10.3390/nano15110782 (PMC12157313; doi:10.3390/nano15110782)

Supplementary Material

Figure S1. Adsorption isotherms at 298 K for (a) AB29, (b) CV, (c) EY, (d) MG, (e) RB, according to Langmuir and Freundlich models.

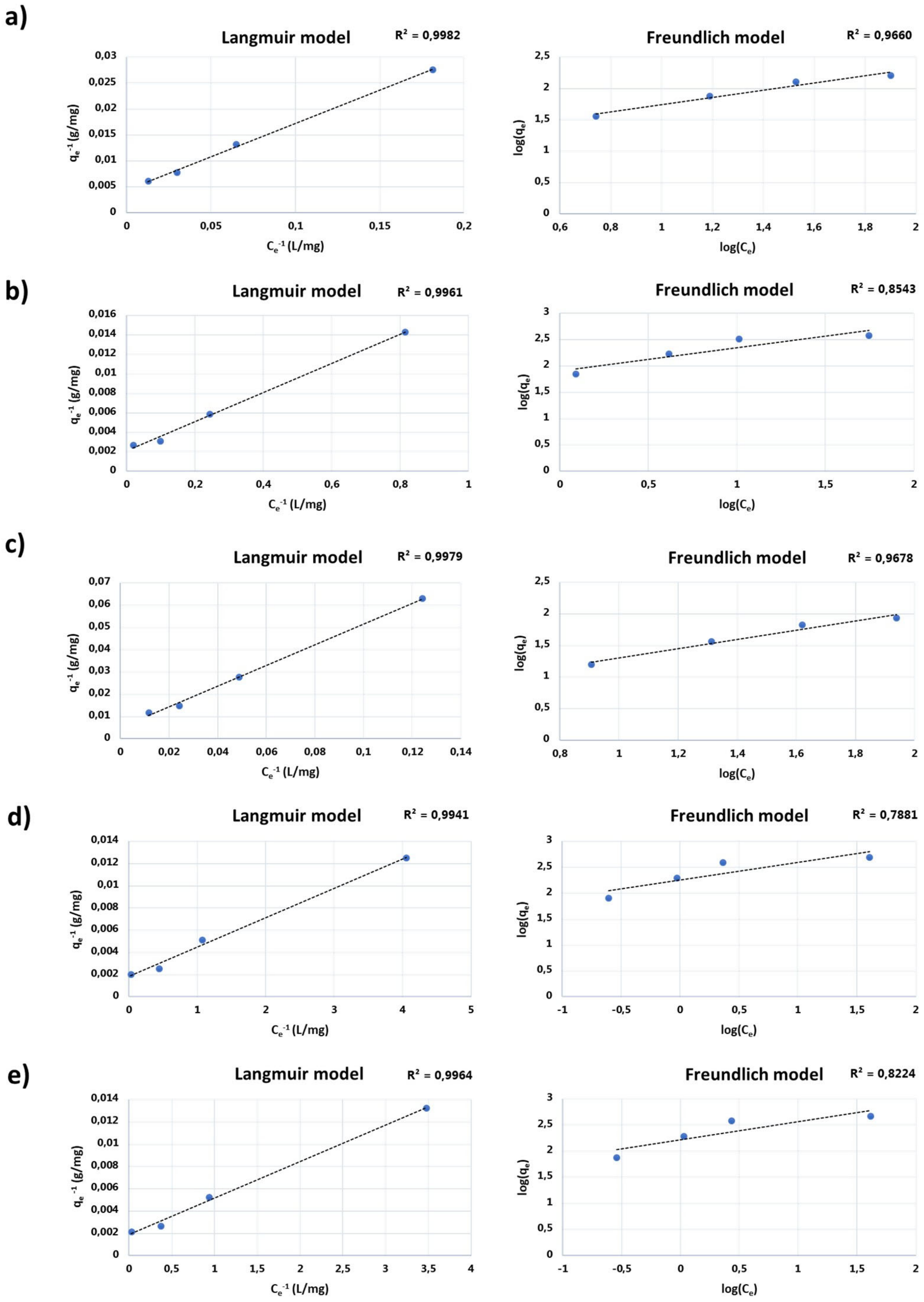

Figure S2. Adsorption isotherms at 313 K for (a) AB29, (b) CV, (c) EY, (d) MG, (e) RB, according to Langmuir and Freundlich models.

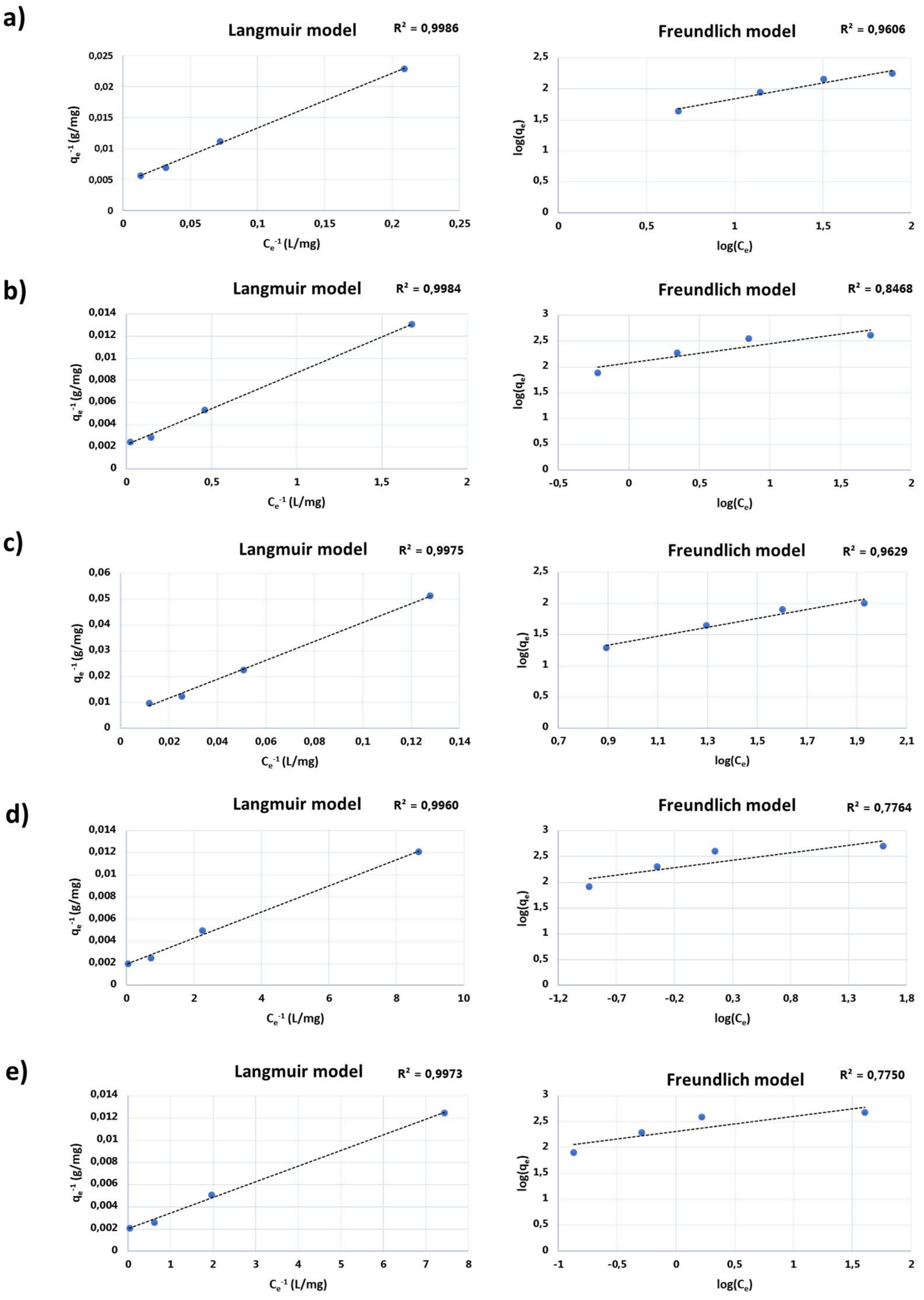

Supplement: Supplementary file 1 [file nanomaterials-15-00782-s001.zip › nanomaterials-3617353-supplementary.pdf]
